# Non-Invasive Determination of Annual Fecal Cortisol, Androstenedione, and Testosterone Variations in a Herd of  Male Asian Elephants (*Elephas maximus*) and Their Relation to Some Climatic Variables

**DOI:** 10.3390/ani11092723

**Published:** 2021-09-17

**Authors:** Paloma Jimena de Andrés, Sara Cáceres, Belén Crespo, Gema Silván, Juan Carlos Illera

**Affiliations:** 1 La Reserva del Castillo de las Guardas, 41898 Sevilla, Spain; 2 Department of Medicine and Surgery, Veterinary Faculty, Complutense University of Madrid, 28040 Madrid, Spain; 3 Department of Physiology, Veterinary Faculty, Complutense University of Madrid, 28040 Madrid, Spain; sacacere@ucm.es (S.C.); belencre@ucm.es (B.C.); gsilvang@vet.ucm.es (G.S.); jcillera@ucm.es (J.C.I.)

**Keywords:** feces, glucocorticoids, adrenal activity, gonadal activity, non-invasive monitoring

## Abstract

**Simple Summary:**

Studies of physiology, animal welfare, and behavior in endangered species are gaining more importance with the aim of contributing to their conservation, and studies that use non-invasive methods for hormonal determinations are especially valuable. In this study, feces were used to assess whether the adrenal and gonadal hormones showed variation in male Asian elephants in a 14-month period and to evaluate whether there were any influences of climatic variables with respect to hormonal secretion. We show here that the use of non-invasive methods to measure the levels of steroid hormones in Asian elephant feces allows us to obtain useful results without having to subject the animals to stressful activity or requiring specific training to obtain the samples. Additionally, this research provides a deeper understanding of endocrine regulation in captive Asian elephants (*Elephas maximus*) in order to enhance reproduction programs in captivity and conserve the species.

**Abstract:**

The measurement of stress and reproductive hormones in wild animal species by non-invasive methods is of special interest. To assess whether the adrenal and gonadal hormones show annual variations in male Asian elephants (*Elephas maximus*) and to evaluate whether there is any influence of climatic variables on hormonal secretion, fecal samples were taken from a herd of 7 Asian elephants over a 14-month period to subsequently determine the concentrations of testosterone (T), androstenedione (A4), and cortisol (C) by a validated immunoassay technique. Data referring to three climatic variables in the place and period of study were collected, namely monthly mean values of temperature, humidity and rainfall. Levels of T and A4 showed two major increases in July (T: 1088.35 ± 131.04 ng/g; A4: 480.40 ± 50.86 ng/g) and October (T: 825.09 ± 31.60 ng/g; A4: 319.96 ± 32.69 ng/g) (*p* < 0.05). Our results show a secretion of fecal androgens dependent on temperature (T and A4), and humidity (T). Male musth was detected during the initial increases of T and A4 levels. The highest concentrations of C were observed in September (156.67 ± 60.89 ng/g) (*p* < 0.05), probably due to the stressful fights that occurred during the musth period. The observed results of the fecal levels of T, A4 and C were similar to those obtained by invasive methods. In conclusion, fecal secretion of the three hormones in these captive male Asian elephants showed variations related in some cases to different weather factors.

## 1. Introduction

Non-invasive techniques are being increasingly used in endangered wildlife populations to objectively measure the animals’ well-being and determine their reproductive status [1,2,3]. Biological samples can be used to perform non-invasive hormone assays, including feces, urine, and saliva [4,5,6]. The use of fecal material to measure stress and reproductive hormones presents several advantages, as this type of sampling can be performed without the need for physical or chemical restraint, both of which create unnecessary stress for the animal and can potentially alter the hormones being measured [7]. Besides, hormone metabolites are quite stable in Asian elephant (*Elephas maximus*) feces [8].

Circannual periodicity in circulating steroid hormones has been found in many mammals including deer, humans, squirrels and horses [9,10,11,12]. However, little is known about the annual secretion of cortisol (C), androstenedione (A4) and testosterone (T) in male Asian elephants as measured by their fecal concentrations. 

Elevations in plasma T levels have been detected in males in musth [13,14,15,16], with the most pronounced levels in older animals [14] or dominant individuals [15]. Similar observations were made in captive elephant bulls [17,18]. Although the principal androgen secreted during musth periods is T and during non-musth periods is A4, both hormones in serum have been shown to fluctuate similarly over a one-year period in an individual elephant [19]. In contrast, there is disagreement regarding C production and whether it increases as a result of musth or not [17,18,20,21].

These studies focus on the role of the reproductive hormones during musth. However, there is a paucity of data regarding the influence of climatic variables on endocrine regulation in male elephants. The influence of seasonality on the reproduction of mammals is a topic of interest [22,23,24,25,26,27]. The breeding season of elephants is not easily defined, however. There is some evidence to suggest that female Asian and African elephants in their natural range areas exhibit seasonal variation in reproductive hormone levels [25,28], but there is no evidence in the literature on male elephants. A recent review of breeding seasonality in wild and captive Asian elephants investigated the influence of many factors, including body condition, photoperiodic signaling, environmental conditions, intraspecific social interactions, and human management on reproductive cycles. Despite the fact that they can reproduce throughout the whole year, there seems to be a moderate seasonal pattern of reproduction, so the authors of that review described them as “long-day breeders” with underlying photoperiodic cueing [24]. This conclusion was reached based on births and conceptions. Therefore, it would be very interesting to elucidate whether there could be any influence of male endocrinology patterns and to determine additional non-photoperiodic triggers of reproduction that might influence the male endocrine regulation.

Cortisol is commonly used as a stress biomarker in many species [2,28,29,30,31,32,33]. For many researchers studying domestic species, the relationship between weather conditions and stress hormones has generated great interest, since variations of climatic variables are recognized as a potential threat to the growth and production of livestock species [29,33]. In elephants, it was initially reported that fecal C concentrations were inversely correlated with rainfall, being significantly higher in free-ranging female African elephants (*Loxodonta Africana*) in the dry season [28]. In contrast, it was reported that fecal glucocorticoid metabolites were positively correlated with rainfall and temperature in a semi-captive population of Asian elephants in Myanmar [30]. Outside their range areas and in zoo settings, there is also evidence for seasonal variation in C in female Asian elephants [31]. Clubb and Mason (2002) [34], in a review of the welfare of zoo elephants in Europe, identified cold and wet climate as a potential cause of poor welfare. Moreover, they suggested that stress could be implicated in low libido in elephants, which eventually affects their fertility, as has been reported in other species [35,36].

The occurrence of estrus synchrony has been reported not only in free-ranging Asian and African elephant populations [37], but also in captive African elephants [38]. Chemical signals such as pheromones are known to play an important role in inter- and intrasexual communication associated with reproduction in many species, and they are also thought to be involved in the estrus synchrony observed in elephants [15,38,39]. Males in musth show temporary gland secretion and dribbling of strongly odoriferous urine, and both fluids are thought to free chemical signals (pheromones). It is known that females prefer musth males for conception [37], but there is a lack of information on the role of male pheromones in male-to-male communication; only some bull-to-bull observations (flehmen responses and conspecific musth secretion) have been reported [15].

Asian elephants are increasingly threatened in the wild. Populations grow well in undisturbed areas, but in some areas, Asian elephants are constantly driven out of their natural habitat and killed in human-elephant conflicts. At the same time breeding success in captivity is low in most countries of the world. In Europe, efforts have been ongoing to build up a self-sustaining population [40]. Much effort must be made to maintain this self-sustainability, which is the most important goal to be achieved by ex situ conservation programs. Ex situ breeding programs are considered essential for global diversity as they maintain captive populations with precious genetic material for global conservation [41]. Additionally, animals in ex situ breeding programs provide us with the opportunity to conduct research in order to increase our scientific understanding of the species.

The aims of this study were as follows: (i) to evaluate whether fecal metabolites of C, A4 and T show variations during a 14-month study period in a group of male Asian elephants, (ii) to assess possible correlations of the assayed hormones with three climatic variables (humidity, rainfall, and temperature), and (iii) to investigate the relationships among the analyzed hormones and between the hormones and the manifestations of musth.

## 2. Materials and Methods

### 2.1. Animals and Collection of Fecal Samples

This study was conducted at La Reserva del Castillo de las Guardas, an Iberian Association of Zoos and Aquaria (AIZA) accredited zoo in Seville, Spain. Fecal samples were obtained from seven Asian elephant bulls (*Elephas maximus*) ranging in age from 6 to 18 years, living as a bachelor herd. During the sampling period, the elephant bulls were kept in one group. No female was present during the sampling period. The animals were kept under standard zoo management [40]. They were maintained in their indoor enclosures until 09:00 and then released into the outdoor enclosures, where they ranged freely in a 40,000 m^2^ meadow until 18:00 throughout the year. At night, each elephant was housed in an individual cage in two buildings, maintaining audio contact. Although hay was available *ad libitum*, a small portion of pellets, fruits and alfalfa was offered twice per day, in the morning (08:30) and when the animals came back from the outdoor enclosures, with *ad libitum* water. All animals were trained and managed using protected-contact by keepers with whom they were familiar for many years. The details of the animals included in the study are shown in Table 1.

Fresh fecal samples were collected from each individual cage at 09:00 on a weekly-biweekly basis over a 14-month period (from October 2015 to November 2016). Samples were always collected at this time to avoid circadian variations in fecal hormone concentrations. Feces were removed directly after defecation in order to avoid cross-contamination with urine or contamination with other fecal samples in the area. The feces were frozen within 1 h of collection and stored individually in ziplock sandwich bags at −20 °C until assayed. In total, 224 samples were collected and analyzed.

The appearance of physical changes in the seven elephants was used to distinguish between the two conditions musth and non-musth. A male was classified as being in musth when at least one of two physical changes, temporal gland swelling or temporal gland secretion, was observed continuously (for more than three days a week) [14]. These changes were recorded by the keeper staff using a standardized check sheet. 

### 2.2. Fecal Extraction and Enzyme Immunoassays for Steroid Hormones

A total of 224 fecal samples from Asian elephants were analyzed for quantification of fecal C, A4, and T levels.

Prior to hormonal analyses, steroid hormones from fecal samples were extracted by using a previously established method in our laboratory [3]. Briefly, frozen fecal samples were dried and pulverized, then 0.2 g of powered feces was extracted by adding 5 mL of 90% ethanol, followed by centrifugation (500× *g*, 20 min) and supernatants were introduced into glass tubes. These supernatants were then boiled for 20 min. Pellets were again processed as previously described. The second supernatants were added to the first set of tubes, dried once more and then resuspended in 1 mL of 100% methanol. The extracts were stored at −20 °C until hormonal analysis. The efficiency of extraction of each steroid hormone from fecal samples was tested by the addition of radiolabeled hormones (3H-cortisol, 3H-androstenedione and 3H-testosterone, 4000–8000 dpm; ICN, CA, USA) to a parallel set of fecal samples prior to extraction.

Steroid hormones (C, T, and A4) from fecal extracts were analyzed by enzyme immunoassays developed in our laboratory. The validation parameters (recovery rates, sensitivity, linearity, intra- and inter-assay coefficients of variation, and parallelism) were assayed as previously reported by Pineiro et al. (2020) [42]. The enzyme immunoassay (EIA) techniques and antibodies used were developed and validated at the Endocrinology Laboratory of the Department of Animal Physiology, Faculty of Veterinary Medicine, Complutense University of Madrid. 

Polyclonal antibodies against cortisol (C9130), testosterone (R156), and androstenedione (C9111) were raised in rabbits. All antibodies were then purified and characterized for cross-reactivity against related steroid hormones. Hormone conjugates cortisol-3HS, androstenedione-·HS and testosterone-3HS were labeled by horseradish peroxidase. All steroids were obtained from Steraloids Inc.

Enzyme immunoassays were performed following the same assay protocol: flat bottomed 96-well polystyrene microtiter plates were coated with 100 μL/well of each purified antibody solution (1:8000 for cortisol and testosterone; 1:5000 for androstenedione) in coating buffer of sodium carbonate (50 mM, pH 9.6) and incubated overnight at 4 °C. Afterwards, non-bound antibodies were removed from the wells by washing plates five times with wash solution (NaCl 150 M/L, Tween 20 0.5 mL/L), inverted, and dried.

Standards were solubilized in ethanol, evaporating the solvent and resolubilizing them in assay buffer (sodium phosphate, 100 mM, pH 7.0, with sodium chloride, 8.7 g/L, and BSA, 1 g/L). The standard curve covered a range between 0 and 1 ng/well and was constructed by using 10 standard solutions: 0.1, 0.5, 1, 5, 10, 50, 100, 500, and 1000 pg/well. Standards and fecal extracts were analyzed in duplicate. For standard curves, each standard was resuspended in 150 µL of diluted conjugates (1:80,000 for cortisol, 1:40,000 for testosterone; and 1:50,000 for androstenedione) and mixed, and 50 µL was pipetted into the wells. For fecal samples, 25 µL of each extract was evaporated and resuspended in 150 µL of diluted conjugate, and 50 µL was pipetted into the wells. Plates were covered and incubated for 2 h at room temperature. Bound/free separation was achieved by emptying plates by inversion and washing them five times with wash solution. To evaluate the amount of labelled hormone bound to the antibody, 100 µL of substrate solution (3,3,5,5-tetramethylbenzidine dihydrochloride) was added to all wells and incubated for 15 min at room temperature, then this reaction was stopped by the addition of 100 µL of 1 M phosphoric acid. Absorbance was read at 450–600 nm in an automatic microplate reader.

Hormone concentrations were calculated by means of software developed for these techniques. Standard-dose response curve was constructed by plotting the binding percentage (B/B0 × 100) against hormone standard concentrations added. Fecal steroid hormone concentrations were expressed as nanograms per gram (ng/g) of dry fecal matter.

### 2.3. Climatic Data

Data on monthly average temperature (°C), total rainfall (L/m^2^) and humidity (%) recorded at the closest weather station to the location of La Reserva during the study (October 2015 to November 2016) were retrieved from the Spanish State Meteorological Agency.

### 2.4. Statistical Study

SPSS 25.0 (IBM Statistical Package for the Social Sciences, USA, 2017) was used for statistical analysis. Fecal steroid hormone concentrations were expressed as ng/g dry fecal matter. These results were expressed as monthly group mean ± SD. Repeated measures analysis was used to assess hormonal variation along the 14-month period of study, and partial η^2^ value was used to test the size effect. The Friedman test for related samples was used to group the data into homogeneous subsets.

The Spearman rank correlation test was used to measure the degree of association between the climatic variables and the hormones assayed and among the hormones. 

Musth condition was recorded when at least one animal in the group showed the external signs previously described. In all statistical comparisons, *p* < 0.05 was accepted as denoting significant difference.

## 3. Results

### 3.1. Climatic Data

Monthly average temperatures varied from the extremes of 10.5 to 28 °C, monthly total rainfall varied from 0 to 204.8 L/m^2^ and humidity varied from 37 to 85%. Temperature peaks were observed in July and August, coinciding with the minimum value for humidity. The lowest temperatures were recorded in January and February, when the humidity was at its maximum (Table 2).

### 3.2. Hormone Concentrations

The sensitivity of the EIA technique was verified by a low limit of detection and was calculated in 10 consecutive assays. The results from low limit detection were as follows: T  =  9.46 pg/well; A4  =  4.67 pg/well; C  =  2.11 pg/well. The assessment of the recovery rates of the conjugate gave the following results: T, 1.1:1; A4, 1.2:1; C, 1.4:1 moles. The recovery of enzyme activity after conjugation was more than 86% in all cases. The precision of T, A4, and C EIAs was determined by calculating the intra- and interassay coefficients of variation (CV%). The results from CV% were as follows: T: intra = 3.1  ±  0.82%, inter = 4.9  ±  0.89%; A4: intra = 5.0  ±  0.94%, inter = 6.2  ±  0.98%; C: intra = 7.0  ±  1.02%, inter = 8.9  ±  1.86%. In order to determine the effects of fecal samples on the standard curves, the standard curves with fecal samples were run in parallel with the standard dose–response curve. Correlations between both standards curves resulted in a good degree of parallelism between them for the hormones studied (T, R2  =  0.82; A4, R2  =  0.81; C, R2  =  0.84). Linearity was demonstrated by performing serial dilutions of a pool of fecal samples (1:1- to 1:32-fold) and comparing them to the percentage of binding inhibition to antibody in the assay (B/B0 percentage), which was linear in the range of 1:1- to 1:3-fold dilution for T, 1:2- to 1:5-fold for A4, and 1:1- to 1:6-fold for C. The addition of exogenous hormone (3.0, 30.0, and 300.0 ng of testosterone, androstenedione, and cortisol per gram of sample) to the pooled fecal samples with high and low hormone concentrations showed a mean recovery of 92% for T, 89% for A4, and 91% for C.

Table 3 summarizes the hormone concentrations obtained over the 14-month period in each sampling month.

#### 3.2.1. Testosterone

**Figure 1:**
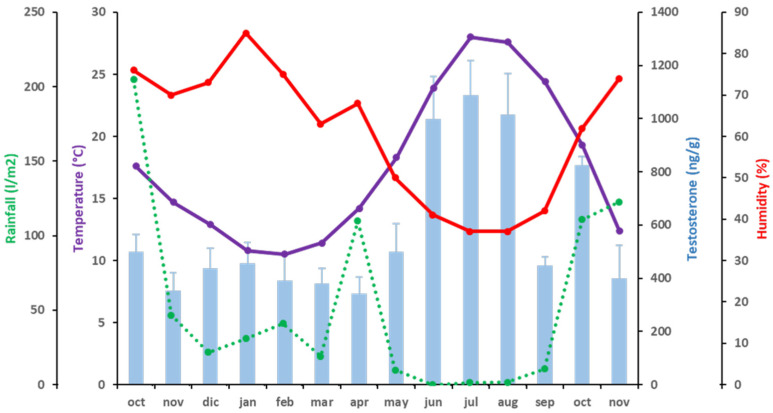
Correlations between T (blue bars) and climatic variables [temperature (purple), rainfall (green) and humidity (red)] during the period of study. A discontinuous line represents independence of the variable.

The mean values of the fecal concentration of T ranged from 340.94 ± 63.82 ng/g dry fecal material, the lowest concentration, observed in April 2016, to 1088.35 ± 131.04 ng/g dry fecal material, the highest concentration, in July 2016. From October 2015 to May 2016, during winter and spring, T levels were below 500 ng/g dry fecal material. Then, two elevations of T levels were observed: the first one was a plateau during June, July, and August 2016, when the level was around 1000 ng/g (*p* < 0.05; partial η^2^ = 0.976), and, after a decrease in September 2016 (446.7 ± 34.72 ng/g dry fecal material) (*p* < 0.05; partial η^2^ = 0.946), there was a smaller, shorter increase in October 2016 (825.09 ± 31.60 ng/g dry fecal material) (*p* < 0.05; partial η^2^ = 0.997). After this peak, T levels again decreased below 500 ng/g dry fecal material in November 2016 (398.76 ± 123.83 ng/g dry fecal material) (*p* < 0.05; partial η^2^ = 0.932). Fecal T concentrations were positively correlated to T° (*p* < 0.01; ρ = 0.723) and H (*p* < 0.01; ρ = −0.563) (Figure 1 and Table 4).

#### 3.2.2. Androstenedione

**Figure 2:**
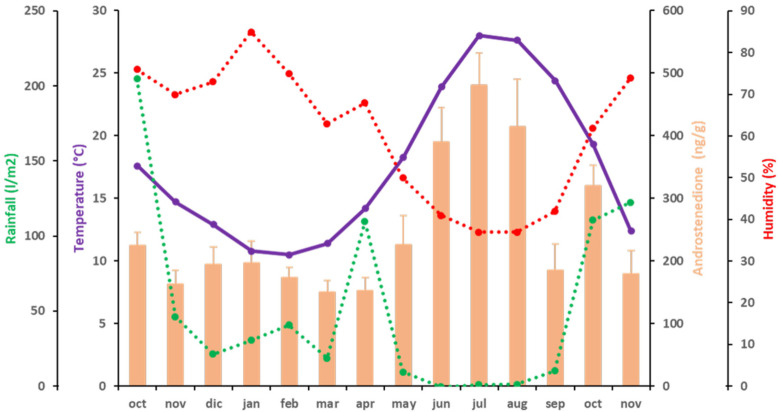
Correlations between A4 (orange bars) and climatic variables [temperature (purple), rainfall (green) and humidity (red)] during the period of study. A discontinuous line represents independence of the variable.

The variations in mean values of A4 fecal concentration were similar to those described for T (Figure 2). The fecal A4 levels of the elephants ranged from 150.69 ± 18.22 ng/g dry fecal material, the lowest concentration, observed in March 2016, to 480.40 ± 50.86 ng/g dry fecal material, the highest concentration, in July 2016. As for T, lower A4 levels were observed from October 2015 to May 2016 (below 300 ng/g dry fecal material). Then, A4 started to increase to a maximum in July 2016 (480.40 ± 50.86 ng/g dry fecal material) (*p* < 0.05; partial η^2^ = 0.893). From August to September 2016, A4 levels again decreased below 300 ng/g dry fecal material (185.83 ng/g dry fecal material) (*p* < 0.05; partial η^2^ = 0.894), then a mild increase was observed in October 2016 (319.96 ± 32.69 ng/g dry fecal material) (*p* < 0.05; partial η^2^ = 0.917). After this peak, A4 levels again decreased below 300 ng/g dry fecal material in November 2016 (179.73 ± 36.52 ng/g dry fecal material) (*p* < 0.05; partial η^2^ = 0.974). Fecal A4 concentrations were positively correlated to T° (*p* < 0.01; ρ = 0.723) (Figure 2 and Table 4). Although A4 and H were not significantly correlated, there was a trend of significance between these two variables (*p* = 0.055; ρ = −0.524) (Figure 2 and Table 4).

#### 3.2.3. Cortisol

**Figure 3:**
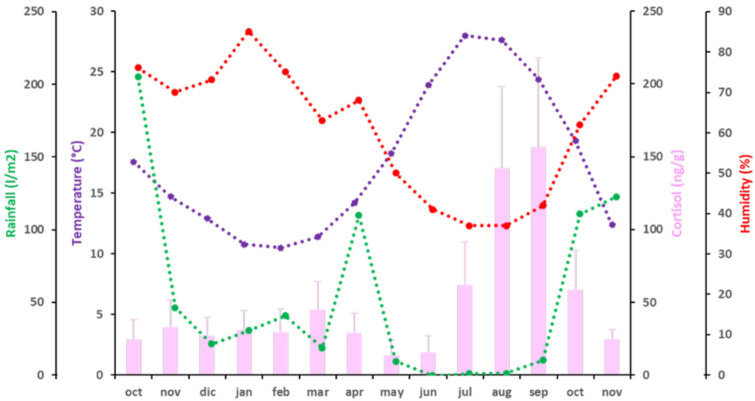
Independence of C (pink bars) and climatic variables [temperature (purple), rainfall (green) and humidity (red)] during the period of study. A discontinuous line represents independence of the variable.

The mean values of C fecal concentration ranged from 13.97 ± 10.53 ng/g dry fecal material, the lowest concentration, observed in May 2016, to 156.67 ± 60.89 ng/g dry fecal material, the highest concentration, in September 2016 (Figure 3). From October 2015 to June 2016, during winter and spring, fecal C levels were below 50 ng/g dry fecal material. Then, C levels started to increase in early summer, to 62.04 ± 29.54 ng/g dry fecal material in July 2016 (*p* < 0.05; partial η^2^ = 0.857). An elevation of C levels above 100 ng/g dry fecal material was observed during August and September 2016, when C levels were around 150 ng/g (*p* < 0.05; partial η^2^ = 0.897). After this plateau, an important decrease in fecal C levels was observed, coinciding with the beginning of autumn in October 2016 (58.59 ± 27.08 ng/g dry fecal material) (*p* < 0.05; partial η^2^ = 0.895), and again below 50 ng/g dry fecal material in November 2016 (24.70 ± 7.2 ng/g dry fecal material) (*p* < 0.05; partial η^2^ = 0.67). Fecal C concentrations were independent of all climatic variables studied (T°, H, and rainfall) (Figure 3 and Table 4).

### 3.3. Correlations

Table 4 summarizes the degrees of association between the climatic variables and the hormones assayed and among the different hormones.

**Figure 4:**
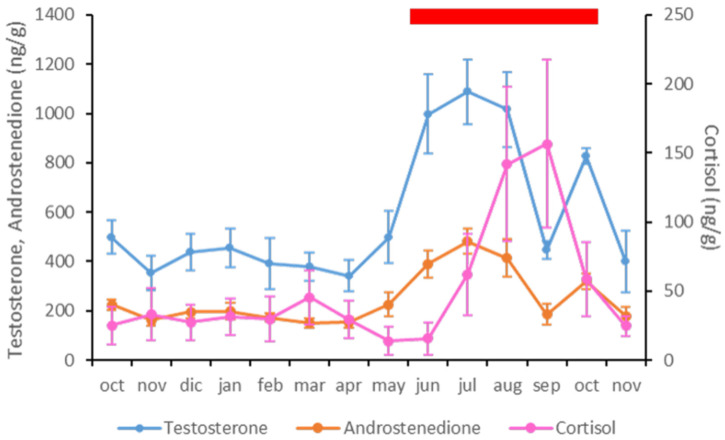
Representation of fecal testosterone (blue), antrostenedione (orange) and cortisol (pink) concentrations in the Asian elephant bulls included in the study. The red rectangle represents the musth period according to the definition used in this study.

Apart from the correlations described above for each hormone with respect to the climatic variables, fecal A4 and T levels were positively correlated, since the variations were similar throughout the study period (*p* < 0.01; ρ = 0.982) (Table 4 and Figure 4). However, there were no correlations between fecal C levels and the other hormones analyzed (Table 4 and Figure 4).

Although it was not an objective of this study, it is interesting to note that humidity was negatively correlated with T° (*p* < 0.01; ρ = −0.84) and positively correlated with rainfall (*p* < 0.01; ρ = 0.748) (Table 4).

### 3.4. Musth Condition

In the 14-month period of the study, the musth condition was observed from June to October 2016 (Figure 4). The strongest intensity of the external signs previously described was observed in all animals in August 2016, except for the youngest animal, a six-year-old male, who never showed any signs of musth during the trial. The animals did not all show the same intensity and duration of musth external signs. However, the musth intensity observed in August 2016 in all the animals led to very aggressive behavior. As a consequence, the daily behavioral routines were sometimes altered, requiring that an individual be kept in a different outdoor enclosure from time to time, in order to avoid physical contact. This enclosure was close enough to permit visual, olfactory, and auditory contact with the other animals in the group. At the end of October 2016, the management routines were completely recovered. At this institution, the musth condition has been always observed during the hottest months of the year (personal communication).

## 4. Discussion

Studies of physiology, animal welfare, and behavior in endangered species are gaining more importance, with the aim of contributing to their conservation, and those that use non-invasive methods for hormonal determination are especially valuable. In this study we investigated for the first time reproductive and stress hormones in a closed group of seven male Asian elephants living together in captivity using noninvasive techniques. Although the main objective of our study was to evaluate the fecal levels of T, A4, and C in the group of elephants, we also wanted to know the influence of climatic variables on these hormones and their relationship with the musth condition. Although the number of animals included in the study seems to be low, this group of Asian elephants was the largest bachelor herd kept in captivity in Europe as of January 2016 [43], and they provided us with valuable data, as they were kept under the same management procedures and the same environmental conditions. Therefore, this study could contribute in some manner to the ex situ conservation and welfare of Asian elephants, since it increases our knowledge of the species´ biology, and our results could contribute to improving the management procedures for elephants in captivity as well as breeding programs.

Our results show that the use of fecal samples to investigate the levels of T, A4, and C is a useful method, as well as simple, as it facilitates sampling by the keeper. The design of the study established the need to take samples within 30 min after elimination, but Wong et al. [8] already established the stability of glucocorticoids in Asian elephant feces for eight hours subjected to different H and T° conditions, so if there would have been a delay from the time of elimination until collection and subsequent freezing, it would not have varied or affected the values obtained in the present study. In fact, this has been demonstrated to be a very valuable method when investigating different hormones in free-ranging elephants [28,44].

This non-invasive technique helped us to determine that the secretion of androgens (T and A4) in this group of male Asian elephants undergoes annual variation dependent on two climatological variables. The levels of T and A4 increased notably in the summer months (June, July and August), with a second increase in October. Lower T and A4 levels were recorded in the winter-spring season. Both hormones showed similar variations, therefore were correlated with each other. A 3.2-times increase from the lowest concentration to the highest was observed in both T and A4. The highest levels were observed when animals showed the musth condition. Similar variations in serum testosterone concentrations were previously observed in three Asian elephant bulls living together in the same facility [16]. Although other authors [19] have indicated that during the absence of musth A4 prevails over T, and this relationship is reversed during the period of musth, our results contradict this observation, since the mean levels of T exceeded those of A4 during the entire study period. This discrepancy may be due to the fact that our results are based on means obtained for the entire herd as a whole, without individual analysis, unlike what was done in the previous study. Furthermore, those authors did not indicate how many animals this finding related to, or how it would have turned out if all animals had been considered.

It is important to note that all of the animals in this study showed synchronization of hormone levels. The six-year-old male, Dimas, who never showed any signs of musth, had levels similar to the others. Male Asian elephants generally reach puberty between 8 and 12 years of age. During this time, the complicated hormonal control system of reproduction is working, but they may not be able to reproduce. It is important to note that environmental conditions can alter the age of puberty in Asian elephants [40]. Others have suggested that while androgen levels increase during musth, they are not necessarily associated with the expression of its signs [45], which could explain the absence of signs of musth in this animal. In addition, males in musth have been shown to emit volatile compounds from the temporal gland and within their urine, which may have an impact on other bulls [15,39]. We think that this chemical communication could be a form of endocrine synchronization that accounts for Dimas’s endocrine levels. Synchronization has also been observed in females [37,38] and in other adult Asian elephant bulls after living together for years [15,16].

It was interesting, once the variations of both hormones were known, to investigate whether there was a climatic influence on their secretion. We found that T and A4 were clearly influenced by the environment. During the hottest months (July and August), the levels of T and A4 showed a large increase, and during the cold season, low levels of both hormones were detected. On the contrary, we observed higher levels of T when humidity was low and low levels when humidity was high. Analyzing these results, we can conclude that T° seems to be the climatic factor that most influences the rate of secretion of these androgens, and humidity also seems to have an influence, although to a lesser extent In India and Myanmar, several studies reported conceptions in Asian elephants to occur mainly in the long-day period in the dry season [46,47,48]. Similarly, in zoo-kept Asian elephants, at latitudes from 43° to 53° N, where most zoo elephants live, more conceptions were recorded from February on, and conceptions peaked in June and again in the beginning of September, with lower reproductive activity from October to January [24]. In wild animals, differences in reproductive rates between seasons may be due to food availability and animal health. However, if similar seasonal reproduction is observed in zoo animals despite the constant provision of resources in captivity, then we must consider that the reproduction of the species is triggered by an additional signal other than the availability of food [22]. Our results show that high temperatures and low humidity levels might act as non-photoperiodic triggers of androgen production in male Asian elephants. On the other hand, a higher quality of semen has been detected in Asian elephants when they show higher serum levels of testosterone [49]. Putting all of this together and given that La Reserva is at a similar latitude (37° N), our results on androgen levels and their relation to the climatic variables included in the study might explain the successful conception during the times when we observed elevated androgen levels. In addition, artificial insemination in elephants is gaining importance not only with chilled, but also frozen semen [50]. Herds of bulls raised in zoos play an important role in breeding programs, since varied genetic material is kept at the same institution. Thus, our results could help to find the most appropriate moment for multiple semen collection, facilitating the work of specialists in the field.

Therefore, our findings are important to consider, so that zoos and rehabilitation centers can improve captive breeding programs for the species, and thus contribute to its long-term conservation. Further, our results allow us to improve not only breeding programs, but also the handling of animals in captivity and the safety of the personnel responsible for their care. Male Asian elephants in musth are very dangerous animals that put people’s lives at risk [51]. For this reason, in recent years, contraceptive methods have been tried to control both the birth rate of the animals and the aggressive manifestations during the musth period, with gonadotropin releasing factor (GnRH) analogue vaccines [52]. Our results indicate that times of highest androgen levels (when they show the greatest aggressiveness) could be predicted, if we take into account local variations in T° and H. Therefore, our results not only could help to determine the most propitious moment for the administration of this vaccine, but also indicate that its effectiveness could be controlled by detecting variations in the concentrations of T and A4 in the fecal matter, as was done in our study.

With respect to C, the highest levels occurred in August and September, coinciding with the end of summer, while low levels occurred between October 2015 and June 2016. Menargues et al. [31] studied saliva C levels for a year in a group of eight Asian elephants between 21 and 46 years of age housed in captivity in a zoo in Alicante (Spain). They found an increase in C concentration between May and October, with the highest levels in September and October and the lowest in April. Our results are similar, although the highest C levels in our study were detected in August and September, and the lowest in May. These small variations could be due to multiple factors that differ between the two studies, such as the sex of the animals, their age, or the location of the institution. However, the most relevant finding is that the trend in the variations of C in both studies is very similar.

On the other hand, our results also showed another (but not significant) increase in C in March 2016 with respect to previous months. An interesting fact that could explain this result is that Easter was celebrated in that month with an influx of visitors. Cortisol has a very significant role in metabolism and in the response to stress, therefore small fluctuations could occur as a consequence of other factors. Previous studies in zoo animals showed an increase in negative social and vigilance behaviors in response to increased visitor numbers [53]. In our study, the increase in the number of visitors could have been a specific stressor for the animals. However, the increase was not significant and C levels decreased the following month, which could indicate that the animals easily adapted to the situation and there was no worsening of their welfare during that period.

Cortisol was not correlated with the other two hormones, or with climatological variables. However, previous studies regarding the influence of rainfall and temperature on C levels in elephants are contradictory [28,30]. So, the influence of different climatic variables with regard to stress on elephants remains unclear. The animals included in this study had been living at the institution for a long time with these climate variations, so C being independent from the climatological variables studied could be indicative of good adaptation to the climate of the place, such that the climate in our study would not act as a stressor. A previous study identified cold and wet climate as a potential cause of poor welfare [34]. In our study, on January and February 2016, the lowest T and highest H levels were recorded, when C levels were low. As the previous study was carried out on all zoos in the European region, they were able to refer to northern countries with more extreme climatic conditions than Spain when reaching this conclusion. We affirm, therefore, that C secretion in our animals was independent of the climatic factors included here. Other factors influencing C secretion, such as hours of light, have been described in the literature [54], but this factor was not considered in our study. The possible influence of other climatic factors on C levels would be very interesting to investigate in depth in the future. In our study, the most stressful factor that could have caused the C increase seems to be the musth condition.

Regarding the observation of this special condition, male musth was manifested in six of the seven elephants between June and October 2016, coinciding with the hottest season, and therefore the months with the highest secretion of T and A4. Even though in September there was a decrease in T and A4 levels, signs of musth were still observed in the group. Musth is manifested by the secretion and swelling of the temporal gland, varying from one animal to another [14], as we observed in our animals. The elevated fecal androgen levels during musth in all males (Figure 4) could be considered as biological validation regarding the reliability of the assay used [15,16,18,55]. This finding coincides with those previously described based on analyses of serum T levels in other male Asian elephants in captivity [17,18] and assays of fecal androgen metabolites in wild Asian elephants [44]. This may be related to the observation that the appearance of the signs of musth may depend on rising androgen levels, while its maintenance may be related to other factors not studied here. When analyzing the relationships between C and the androgens studied, we did not find any significant correlation. It is interesting to note that the highest rise in C levels occurred in August, which was the month of greatest intensity in manifestations of musth. There is some controversy as to whether musth by itself is a stressful condition for the male elephant, both in the wild [44,55] and in captivity [7,16,18,45]. Some authors established that the values of C and androgens are independent of each other [44], while others found a clear association between increased androgens and C in animals exhibiting musth [16,18,56]. These differences can be attributed to several factors such as environmental conditions (captive versus wild), the measurement of circulating hormones in serum samples versus excreted steroids in feces or saliva, and differences in the technical procedures [16,44]. Our results seem to indicate that the C variations in the 14-month study period may be slightly altered by stressors such as an increase in the visiting public or fights that occur because of the musth period. With these results, we cannot be sure that the highest increase in C levels was a direct consequence of musth, since in our animals´ signs of musth began two months before the peak of C. On the other hand, the major increase in visitors normally occurs in August, but in our study, C levels were higher in September 2016, when the number of visitors had decreased, than in August 2016, Furthermore, it is interesting that the highest C levels coincided with a decrease in androgen levels, in September. On the contrary, the decrease in C in October coincided with the second elevation in androgen levels. This could be due to high concentrations of glucocorticoids, which can have harmful effects on the animal, such as immunosuppression or a loss of reproductive function. Since stress decreases secretion of GnRH in the hypothalamus and other of sex steroids in the gonads, a peak C level would be reflected in a decrease in the androgen level [17], as was observed in our study. In addition, since higher stress can also cause decreased libido in elephants [34], we propose monitoring fecal C levels so as to find the most suitable time for breeding or semen collection.

## 5. Conclusions

This study shows that in a group of captive male Asian elephants (*Elephas maximus*), the production of both T and A4 followed annual variation dependent on external factors such as temperature (T and A4) and humidity (T), with the highest levels observed in the hottest season, which should be taken into account for the success of captive breeding programs for the Asian elephant. However, in this group the production of C showed variation independent of external factors of temperature, humidity and rainfall, with a slight increase in production observed in March and a higher peak in September. This may have been influenced to some extent by other external stressors, but not directly by the levels of T or A4. Musth is a phenomenon that produces physical and behavioral changes in male Asian elephants and occurs initially because of increased levels of T and A4, although they do not seem to be the only endocrine factors on which it depends.

## Figures and Tables

**Table 1:** Details of age, parents, place of birth, and time in the institution of the seven male captive Asian elephants (*Elephas maximus*) from La Reserva del Castillo de las Guardas.

Name of Elephant	Age as on 2016 (Years)	Name of Father	Name of Mother	Place of Birth	Period of Time in La Reserva as on 2016 (Years)
**Tse Pyang**	18	Naing Thein	Thiha Phyu	Emmen	13
**Aung Si**	14	Naing Thein	Thiha Phyu	Emmen	9
**Kan Kaung**	14	Naing Thein	Yu Zin	Emmen	3
**Than Myan**	14	Naing Thein	Htoo Yin Aye	Emmen	3
**Toomai**	12	Emmet	Azizah	Whipsnade	5
**Hunt Bwe**	10	Radza	Swe San Thay	Emmen	5
**Dimas**	6	Ankhor	Cynthia	Berlin TP	2

**Table 2:** Monthly average values of climate variables included [temperature (°C), rainfall (L/m2) and humidity (%)] in the 14-month period of study in La Reserva retrieved from the Spanish State Meteorological Agency.

Month ^1^	Temperature (°C)	Rainfall (L/m^2^)	Humidity (%)
**Oct 15**	17.6	204.8	76
**Nov 15**	14.7	46.8	70
**Dec 15**	12.9	21.8	73
**Jan 16**	10.8	31	85
**Feb 16**	10.5	41.2	75
**Mar 16**	11.4	19	63
**Apr 16**	14.2	109.8	68
**May 16**	18.3	10	50
**Jun 16**	23.9	0	41
**Jul 16**	28	1.4	37
**Aug 16**	27.6	1.6	37
**Sep 16**	24.4	10.6	42
**Oct 18**	19.3	110.8	62
**Nov 16**	12.4	122.8	74

^1^ Oct (October), Nov (November), Dec (December), Jan (January), Feb (February), Mar (March), Apr (April), Jun (June), Jul (July) Aug (August), Sep (September); 15 (2015), 16 (2016).

**Table 3:** Mean ± SD in feces T, A4, and C concentrations expressed as ng/g of dry fecal material analyzed by EIA for the group of 7 elephants during the 14-month collecting trial.

Month ^1^	T (ng/g)	A4 (ng/g)	C (ng/g)
**Oct 15**	498.92 ± 65.83	224.33 ± 21.59	25.12 ± 13.42
**Nov 15**	352.81 ± 68.57	162.66 ± 23.00	33.09 ± 18.81
**Dec 15**	437.10 ± 74.72	195.05 ± 27.50	27.26 ± 12.85
**Jan 16**	455.53 ± 77.88	197.38 ± 33.93	31.41 ± 13.37
**Feb 16**	391.15 ± 102.06	173.30 ± 16.55	29.82 ± 16.03
**Mar 16**	379.70 ± 57.79	150.69 ± 18.22	45.37 ±19.40
**Apr 16**	340.94 ± 63.82	152.66 ± 21.22	29.43 ± 13.49
**May 16**	499.00 ± 105.99	225.85 ± 47.10	13.97 ± 10.53
**Jun 16**	997.34 ± 161.44	389.54 ± 54.41	15.62 ± 11.56
**Jul 16**	1088.35 ± 131.04	480.40 ± 50.86	62.04 ± 29.55
**Aug 16**	1015.11 ± 153.50	414.16 ± 75.18	141.92 ± 55.79
**Sep 16**	446.71 ± 34.72	185.83 ± 41.60	156.67 ± 60.89
**Oct 16**	825.09 ± 31.60	319.96 ± 32.69	58.59 ± 27.09
**Nov 16**	398.76 ± 123.84	179.74 ± 36.52	24.70 ± 7.21

^1^ Oct (October), Nov (November), Dec (December), Jan (January), Feb (February), Mar (March), Apr (April), Jun (June), Jul (July) Aug (August), Sep (September); 15 (2015), 16 (2016).

**Table 4:** Spearman correlation coefficients between the variables of the study [temperature (°C), rainfall (L/m2), humidity (%), testosterone (T) (ng/g), androstenedione (A4) (ng/g), and cortisol (C) (ng/g)] (N = 14).

		Temperature (°C)	Rainfall (L/m^2^)	Humidity (%)	T (ng/g)	A4 (ng/g)	C (ng/g)
**Temperature (°C)**	**Correlation value (ρ value)**		−0.508	−0.84 *	0.723 *	0.723 *	0.349
	***p* value**		0.064	<0.01	<0.01	<0.01	0.221
**Rainfall (L/m^2^)**	**Correlation value (ρ value)**	−0.508		0.748 *	−0.525	−0.477	−0.191
	***p* value**	0.064		<0.01	0.054	0.085	0.513
**Humidity (%)**	**Correlation value (ρ value)**	−0.84 *	0.748 *		−0.563 *	−0.524	−0.383
	***p* value**	<0.01	<0.01		0.036	0.055	0.177
**T (ng/g)**	**Correlation value (ρ value)**	0.723 *	−0.525	−0.563 *		0.982 *	0.13
	***p* value**	<0.01	0.054	0.036		<0.01	0.659
**A4 (ng/g)**	**Correlation value (ρ value)**	0.723 *	−0.477	−0.524	0.982 *		0.068
	***p* value**	<0.01	0.085	0.055	<0.01		0.817
**C (ng/g)**	**Correlation value (ρ value)**	0.349	−0.191	−0.383	0.13	0.068	
	***p* value**	0.221	0.513	0.177	0.659	0.817	

The asterisk symbol (*) denotes significant correlation.

## Data Availability

The data that support the findings of this study are available from the corresponding author upon reasonable request.
